# Retraction: Barriers to access to care in the implementation of telemedicine in public hospitals in Southern Ethiopia: A phenomenological qualitative study

**DOI:** 10.1371/journal.pone.0362243

**Published:** 2026-11-06

**Authors:** The PLOS One Editors

The *PLOS One* Editors retract this article [1] due to concerns about compliance with the PLOS Human Subject Research policy.

Specifically, concerns were identified for the validity and integrity of the ethical approval documentation provided for editorial review. PLOS attempted to contact Wolaita Sodo University regarding the ethical approval of this study, but did not receive a response.

GNB did not respond to the final editorial decision. BAA, CKG, TEK, WMM, and ADW either did not respond directly or could not be reached.

In addition, concerns were identified regarding similarities in wording between participant responses in S3 File in [1]. The corresponding author stated that S3 File includes the original verbatim quotes from participants, but that a small fraction of the content was paraphrased to enhance clarity and readability. Due to the above ethical approval concerns, PLOS did not investigate this matter further.
